# Protein citrullination and NET formation do not contribute to the pathology of A20/TNFAIP3 mutant mice

**DOI:** 10.1038/s41598-023-45324-8

**Published:** 2023-10-21

**Authors:** Karel F. A. Van Damme, Pieter Hertens, Arne Martens, Elisabeth Gilis, Dario Priem, Inge Bruggeman, Amelie Fossoul, Jozefien Declercq, Helena Aegerter, Andy Wullaert, Tino Hochepied, Esther Hoste, Lieselotte Vande Walle, Mohamed Lamkanfi, Savvas N. Savvides, Dirk Elewaut, Bart N. Lambrecht, Geert van Loo

**Affiliations:** 1 VIB, Center for Inflammation Research, Technologiepark 71, 9052 Ghent, Belgium; 2 Department of Internal Medicine and Pediatrics, Ghent University, 9052 Ghent, Belgium; 3 Department of Biomedical Molecular Biology, Ghent University, 9052 Ghent, Belgium; 4 Department of Rheumatology, Ghent University Hospital, 9000 Ghent, Belgium; 5 Laboratory of Proteinscience, Proteomics and Epigenetic Signalling (PPES), Department of Biomedical Sciences, University of Antwerp, 2610 Antwerp, Belgium; 6 Department of Biochemistry and Microbiology, Ghent University, 9052 Ghent, Belgium; 7 Department of Pulmonary Medicine, Erasmus MC, Rotterdam, The Netherlands

**Keywords:** Rheumatoid arthritis, Inflammatory diseases, Cell signalling

## Abstract

A20 serves as a critical brake on NF-κB-dependent inflammation. In humans, polymorphisms in or near the *TNFAIP3/A20* gene have been linked to various inflammatory disorders, including systemic lupus erythematosus (SLE) and rheumatoid arthritis (RA). Experimental gene knockout studies in mice have confirmed A20 as a susceptibility gene for SLE and RA. Here, we examine the significance of protein citrullination and NET formation in the autoimmune pathology of A20 mutant mice because autoimmunity directed against citrullinated antigens released by neutrophil extracellular traps (NETs) is central to the pathogenesis of RA and SLE. Furthermore, genetic variants impairing the deubiquitinase (DUB) function of A20 have been shown to contribute to autoimmune susceptibility. Our findings demonstrate that genetic disruption of A20 DUB function in A20 *C103R* knockin mice does not result in autoimmune pathology. Moreover, we show that PAD4 deficiency, which abolishes protein citrullination and NET formation, does not prevent the development of autoimmunity in A20 deficient mice. Collectively, these findings provide experimental confirmation that PAD4-dependent protein citrullination and NET formation do not serve as pathogenic mechanisms in the development of RA and SLE pathology in mice with A20 mutations.

## Introduction

The anti-inflammatory protein A20 (also known as Tumor Necrosis Factor alpha-induced protein 3, TNFAIP3) acts as a key player in the termination of NF-κB signaling. A20 also protects cells from death, independently of NF-κB regulation^1,2^. Mutations and polymorphisms in the *A20/TNFAIP3* gene have been associated with a plethora of (auto)inflammatory and autoimmune pathologies, including systemic lupus erythematosus (SLE), rheumatoid arthritis (RA) and A20 haploinsufficiency (HA20)^3–7^. Experimental studies in mice have demonstrated that mice with myeloid-specific A20 knockout develop spontaneous polyarthritis, which resembles rheumatoid arthritis in humans^8^. This arthritis development is driven by the necroptotic death of A20 deficient macrophages and the subsequent release of the pro-inflammatory cytokine IL-1β^9,10^. Similarly, mice with dendritic cell (DC)-specific A20 knockout develop multi-organ inflammation with lymphosplenomegaly, myeloid expansion, and spontaneous lymphocyte activation, resembling the pathology observed in SLE^11^. These findings provide experimental evidence supporting *A20* as a susceptibility gene for both RA and SLE in humans.

In a recent study, Odqvist et al. proposed a mechanism suggesting that increased PAD4-dependent protein citrullination and neutrophil extracellular trap (NET) formation contribute to the development of anti-cyclic citrullinated peptide (anti-CCP) antibodies and autoimmune pathology in RA and SLE patients with A20 DUB-domain mutations^12^. Protein citrullination is a post-translational modification catalysed by the enzyme peptidyl arginine deiminase 4 (PAD4), which converts arginine into citrulline on target proteins^13^. Citrullination of histones plays a role in NET formation, a process by which neutrophils release chromatin filaments coated with citrullinated histones and antibacterial proteins to trap pathogens^14,15^. Citrullination is associated with autoimmunity and has been implicated in the pathogenesis of RA, in which it is linked to the production of autoantibodies against citrullinated self-epitopes^16–18^. Similarly, in SLE, PAD4-dependent citrullination and NET formation have been reported to promote autoantibody formation against nuclear antigens and induce the release of type I IFNs from plasmacytoid DCs^18–20^. Notably, necroptosis has recently been shown to activate PAD4, resulting in histone hypercitrullination and NET formation^21^. Additionally, a genetic mutation in the catalytic A20 deubiquitinase (DUB) domain has been found to upregulate *Padi4,* leading to increased protein citrullination and NET formation. These findings suggest that citrullination and NET formation may serve as disease-driving mechanisms in the pathology of RA and SLE in A20 mutant mice^12^.

Here, we aimed to investigate the in vivo significance of PAD4-dependent protein citrullination and NET formation in the autoimmune pathology of A20 mutant mice.

## Results

### A20 DUB mutation does not induce NET formation or autoimmune pathology

Polymorphisms in *TNFAIP3,* the gene encoding A20, are among the most frequently reported risk alleles in RA and SLE ^3–6^. In mice, A20 deficiency in myeloid cells (in *A20*^myel-KO^ mice) or in DCs (in *A20*^DC-KO^ mice) were shown to trigger development of RA or SLE-like pathology, respectively^8,9,11^. In a recent study, Odqvist et al. demonstrated that a mutation in the A20 DUB domain causes upregulation of *PADI4*, leading to increased protein citrullination and extracellular trap formation, suggesting that citrullination and NETs act as upstream pathogenic mechanisms driving RA and SLE autoimmune pathology in patients with A20 mutations^12^.

**Figure 1:**
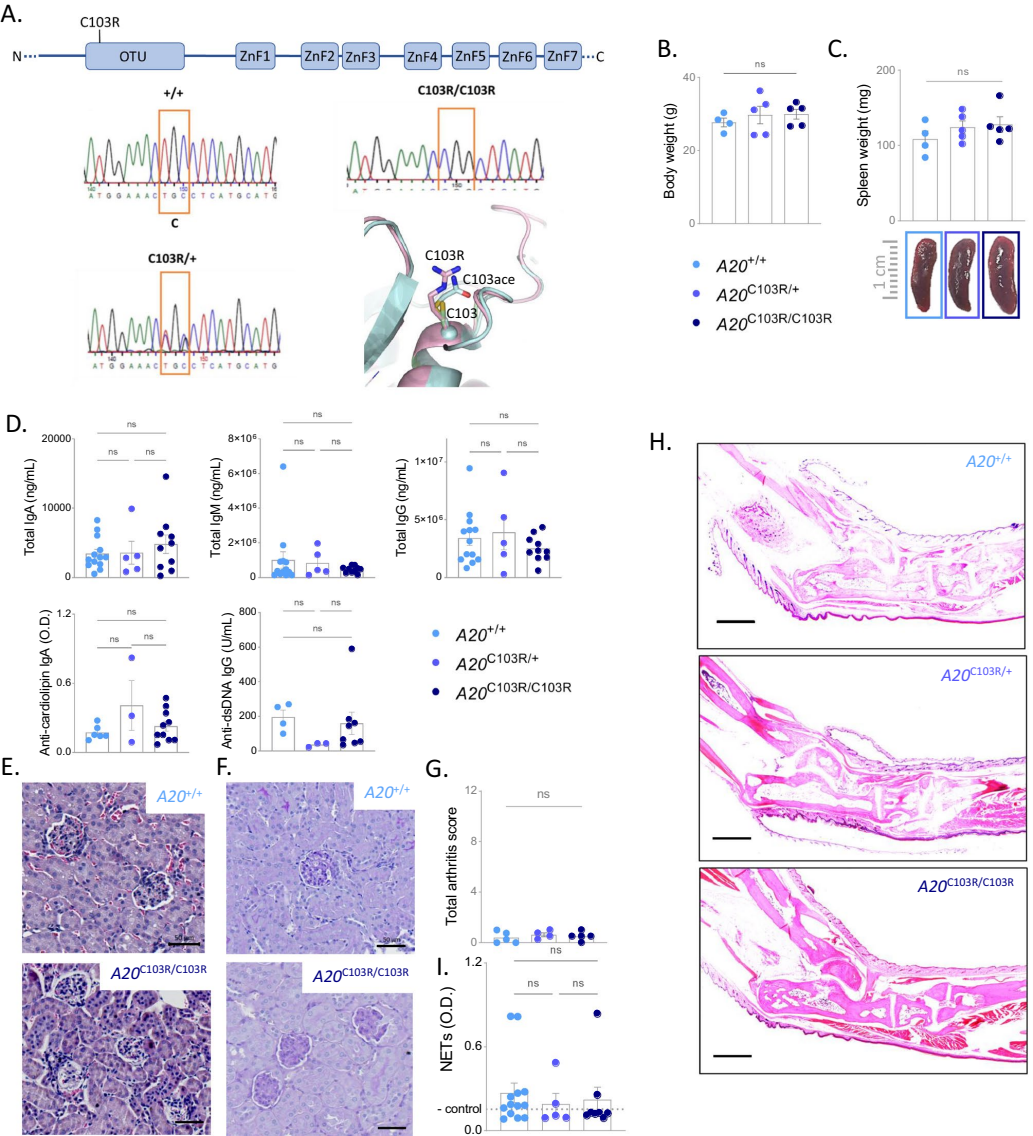
A20 DUB mutation does not induce NET formation or autoimmune pathology. (**A**) Generation of A20^C103R^ knockin mutation in mice using CRISPR/Cas9 technology. A20 domain structure with indication of C103R mutation in OTU deubiquitinase (DUB) domain (upper). Sanger sequencing of wild-type (+ / +), heterozygous (C103R/ +) and homozygous (C103R/C103R) clone (lower). Structural analysis of A20 DUB carrying the C103R mutation (pink, C103R), and comparison with the wildtype A20 DUB structure (cyan, C103) versus acetamidylated A20 DUB at C103 (cyan, C103ace). (**B**, **C**) Body weight (**B**) and spleen weight (**C**) of 25–30 week old wild-type (A20^+/+^), A20^C103R/+^ and A20^C103R/C103R^ mice. Each dot represents one mouse. Data are expressed as mean ± s.e.m. (**D**) Total IgA, IgM, IgG, anti-cardiolipin-IgA, and anti-dsDNA-IgG and neutrophil extracellular trap (NET) concentrations in serum of 25–30-week old mice. (**E–F**) Histological images of haematoxylin and eosin-stained (**E**) and PAS-stained (**F**) kidney sections of 25–30-week old mice, showing normal glomerular architecture and cellularity, and absence of granulomas, tubulo-interstitial atrophy, or vascular changes. Scalebar, 50 µm. (**G**) Histological scores for mice with the indicated genotypes (25–30 weeks). The arthritis was scored at the Achilles tendon (infiltrate) and the synovio-entheseal complex (SEC, exudate), each ranging from 0 (normal) to 3 (severely inflamed). Dots in the graphs indicate individual mice and data are expressed as mean ± s.e.m. (**H**) Histological images of haematoxylin and eosin-stained ankle joints of mice with the indicated genotypes. No signs of an arthritis-like phenotype can be observed in A20^C103R/C103R^ mice. Pictures are representative for 4–5 biologically independent mice for each genotype. Scalebar, 1000 µm. (**I**) Neutrophil Extracellular Traps (NETs) in serum of wild-type (A20^+/+^), A20^C103R/+^ and A20^C103R/C103R^ mice. Each dot represents one mouse. Data are expressed as mean ± s.e.m. n.s., non-significant.

To address the functional consequence of an A20 DUB inactivation in the development of autoimmunity in vivo*,* we generated a knock-in mouse line mutated in the deubiquitinase function of A20 by substituting the active site cysteine (C) residue with an arginine (R) (A20^C103R^) (Fig. 1A). The scientific rationale to develop a A20^C130R^ mouse line and not a A20^C103A^, which has been used in previously published research^22–24^, is based on a recent study that clearly showed that a C-to-A substitution in a DUB dramatically augments its affinity for ubiquitin^25^. Considering that A20 exerts its anti-inflammatory effects mainly through its role as a ubiquitin-binding protein^26^, the strategy of using a C-to-A mutation could result in potential confounding effects associated with alterations in ubiquitin-binding capabilities unrelated to the loss of DUB activity. Our structural analysis also showed that a C103R mutation in A20 would sterically block access to the A20 substrate binding site without affecting the structural integrity of A20 and its active site (Fig. 1A). Direct support for this analysis is provided by the crystal structure of A20 modified by iodoacetamide at position C103^27^, which illustrates that acetamidylation of C103 leads to a structural adduct that closely mimics how an arginine mutation at C103 would structurally project into the A20 DUB active site (Fig. 1A).

Bone marrow-derived macrophages (BMDMs) from A20^C103R/C103R^ knock-in mice show normal NF-κB, p38 and JNK signaling compared with control BMDMs in response to lipopolysaccharide (LPS) and TNF (supplementary figure S2A–C). In addition, we addressed NF-κB and MAPK signaling in TNF-stimulated mouse embryonic fibroblasts (MEFs) that express either wild-type A20, or A20 with a C103R or with a C103A mutation. Similar to our observations in BMDMs, A20^C103R^ expressing MEFs, as well as A20^C103A^ MEFs, have normal NF-κB, p38 and JNK signaling, similar to what is observed in wild-type MEFs (supplementary figure S2D–E), as has been shown before for A20^C103A^ cells^12,22^.

Homozygous A20^C103R/C103R^ mice develop normally, show no signs of inflammation, and have normal numbers of all major immune cell subsets (Fig. 1B–D and supplementary figure S2A–F). Notably, A20^C103R/C103R^ mice do not develop an inflammatory or autoimmune disease, and they do not display any arthritis-like phenotype or SLE-like pathology, even in advanced age (Fig. 1E–H, supplementary figure S1G–J), in contrast to *A20*^myel-KO^ or *A20*^DC-KO^ mice^8,11^.

Finally, no increased neutrophil extracellular trap (NET) formation or upregulated expression of PAD4 by neutrophils could be observed in A20^C103R/C103R^ mice, in contrast to what was previously shown^12^ (Fig. 1I and supplementary figure S3).

### PAD4 deficiency does not prevent SLE pathology in DC-specific A20-deficient mice

A20 has previously been demonstrated to regulate dendritic cell (DC) activation to maintain immune homeostasis. Consequently, mice with DC-specific A20 knockout develop a severe inflammatory pathology reminiscent of SLE^11^. To investigate the contribution of PAD4-dependent protein citrullination and NET formation to the SLE phenotype observed in DC-specific A20 deficient mice, a new mouse line with a knockout allele for *Padi4* was generated via CRISPR/Cas9 targeting (supplementary figure S4A). PAD4 knockout mice are phenotypically normal and exhibit comparable levels of neutrophils to wild-type mice (data not shown). Western blot analysis confirmed the expression of PAD4 in wild-type (PAD4^+/+^) and PAD4^+/−^ neutrophils, but not in PAD4^−/−^ neutrophils (supplementary figure S4B). Furthermore, immunostaining for citrullinated histone-3 (Cit H3), a well-established marker for NETs, confirmed the absence of NETs in PAD4^−/−^ neutrophils stimulated with phorbol myristate acetate (PMA) (supplementary figure S4C).

**Figure 2:**
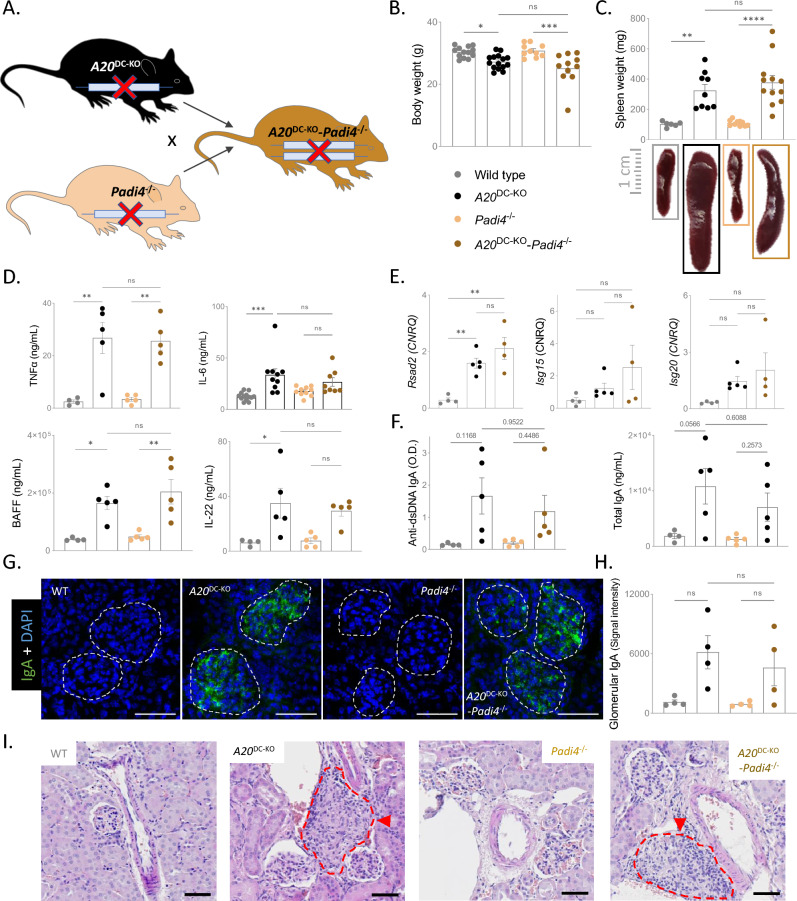
PAD4 deficiency does not rescue DC-specific A20 deficient mice from developing SLE pathology. (**A**) Breeding scheme to generate *A20*^DC-KO^ and *A20*^DC-KO^*Padi4*^−/−^ mice. (**B**, **C**) Body weight (**B**) and spleen weight (**C**) of 25–30 week old wild-type, *A20*^DC-KO^, *Padi4*^−/−^ and *A20*^DC-KO^*Padi4*^−/−^ mice. Each dot represents one mouse. Data are expressed as mean ± s.e.m. (**D**) Serum levels of TNF, IL-6, IL-22 and BAFF in indicated genotypes. (**E**) Quantitative PCR results for interferon-stimulated genes on whole spleen of mice with indicated genotypes. (**F**) Serum auto-antibody concentrations on ELISA in mice with indicated genotypes. (**G**) Representative immunofluorescent image of glomerular IgA deposition per genotype. White dotted circles indicate the glomeruli. (**H**) Quantification of the glomerular mean fluorescent signal for IgA. (**I**) Representative hematoxylin and eosin-stained kidneys of mice with the indicated genotypes, showing extensive perivascular infiltrates in both *A20*^DC-KO^ and *A20*^DC-KO^*Padi4*^−/−^ mice. Each dot represents one mouse. Data are expressed as mean. Each dot represents a biologically independent mouse. *p < 0.05; **p < 0.01; ***p < 0.001; ****p < 0.0001. Scalebar, 50 µm. CNRQ = Calibrated Normalized Relative Quantity. Results are representative of two independent experiments.

DC-specific A20 knockout mice (*A20*^FL/FL^*CD11c*-Cre or *A20*^DC-KO^) were crossed with *Padi4* deficient mice (Fig. 2A), and development of multi-organ inflammation in the presence or absence of protein citrullination was assessed. Both *A20*^DC-KO^ and *A20*^DC-KO^-*Padi4*^−/−^ mice exhibit reduced body weight, splenomegaly (Fig. 2B, C), and elevated levels of inflammatory cytokines in the serum (Fig. 2D). Interestingly, the absence of NETs did not diminish the overexpression of interferon-stimulated genes in the spleen (Fig. 2E). Auto-antibody levels against dsDNA and cardiolipin are comparable between *Padi4*-sufficient and *Padi4*-deficient mice, as well the deposition of glomerular immune complexes, predominantly IgA (Fig. 2F–H). Despite glomerular antibody deposition in *A20*^DC-KO^ mice, irrespective of PAD4 expression, significant urinary protein loss is not observed. Furthermore, no differences in the number of myeloid cells, activated T cells, or the levels of circulating neutrophil extracellular traps were found between *A20*^DC-KO^ and *A20*^DC-KO^-*Padi4*^−/−^ mice (supplementary figure S5). Histological examination of kidney sections via H&E and Periodic acid-Schiff (PAS) staining reveals extensive perivascular infiltrates and subtle glomerulomegaly in both *A20*^DC-KO^ and *A20*^DC-KO^-*Padi4*^−/−^ mice (Fig. 2I). However, immunostaining of the kidneys of these mice does not reveal colocalization of DAPI, MPO and H3citr, which are characteristic for NETs (supplementary figure S6).

Together, these data demonstrate that protein citrullination and NET formation do not serve as pathogenic mechanisms driving SLE pathology in DC A20-deficient mice.

### PAD4 deficiency does not prevent RA pathology in myeloid-specific A20-deficient mice

**Figure 3:**
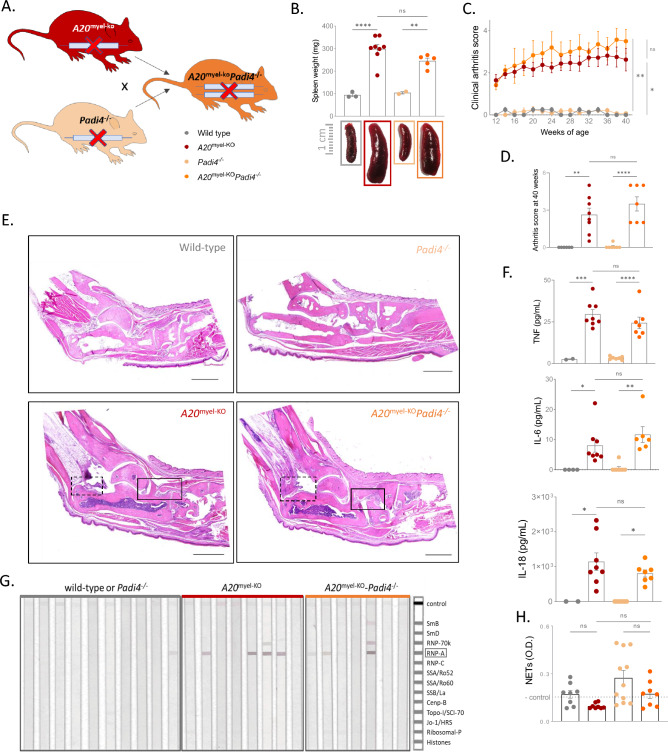
PAD4 deficiency does not rescue myeloid-specific A20 deficient mice from developing RA pathology. (**A**) Breeding scheme to generate *A20*^myel-KO^ and *A20*^myel-KO^*Padi4*^−/−^ mice. (**B**) Spleen weight of 25–30 week old wild-type, *A20*^myel-KO^, *Padi4*^−/−^ and *A20*^myel-KO^*Padi4*^−/−^ mice, with representative picture. Each dot represents one mouse. Data are expressed as mean ± s.e.m. (**C**) Biweekly clinical arthritis scores of the ankles of wild-type, *A20*^myel-KO^, *Padi4*^−/−^ and *A20*^myel-KO^*Padi4*^−/−^ mice. Data are expressed as mean ± s.e.m. ***p < 0.001 (REML analysis). (**D**) Graphs depicting histological scores for mice with the indicated genotypes (25–30 weeks). The arthritis was scored at the Achilles tendon (infiltrate) and the synovio-entheseal complex (SEC, exudate), each ranging from 0 (normal) to 3 (severely inflamed). Total arthritis score is the sum of the 3 individual scores. Dots in the graphs indicate individual mice and data are expressed as mean ± s.e.m. (**E**) Histological images of hematoxylin and eosin-stained ankle joints of mice with the indicated genotypes, demonstrating periarticular inflammation and infiltration of mononuclear cells (insert), as well as the infiltrate in the SEC (dashed insert) in *A20*^myel-KO^ and *A20*^myel-KO^*Padi4*^−/−^ mice. Pictures are representative for 4–5 biologically independent mice for each genotype. Scalebar, 1000 µm. (**F**) Levels of IL-6, TNF and IL-18 in serum of wild-type, *A20*^myel-KO^, *Padi4*^−/−^ and *A20*^myel-KO^*Padi4*^−/−^ mice. Each dot represents a biologically independent mouse. *p < 0.05; **p < 0.01; ***p < 0.001; ****p < 0.0001. (**G**) Line Immunoassay (LIA) of 25-week-old wild-type, *A20*^myel-KO^, *Padi4*^−/−^ and *A20*^myel-KO^*Padi4*^−/−^ mice. Each lane represents an individual mouse. (**H**) Neutrophil Extracellular Traps (NETs) in serum of wild-type, *A20*^myel-KO^, *Padi4*^−/−^ and *A20*^myel-KO^*Padi4*^−/−^ mice. Each dot represents a biologically independent mouse. n.s., non-significant.

A20 deficiency in myeloid cells leads to spontaneous development of a severe and destructive polyarthritis with many features of RA^8–10^. To investigate the contribution of NETs to RA pathology in myeloid-specific A20 knockout mice (*A20*^FL/FL^*LysM*-Cre or *A20*^myel-KO^), these mice were crossed with *Padi4* deficient mice (Fig. 3A), and development of autoimmunity was evaluated. Both *A20*^myel-KO^ and *A20*^myel-KO^-*Padi4*^−/−^ mice display normal body weight (data not shown) but exhibit severe splenomegaly (Fig. 3B) along with arthritis pathology (Fig. 3C, D). Histological examination of ankle joints reveals pronounced synovial and periarticular inflammation, characterized by mononuclear cell infiltration, as well as cartilage and bone destruction (Fig. 3E). Additionally, elevated levels of inflammatory cytokines TNF, IL-6 and IL-18, along with increased serum antibody levels reminiscent of an autoimmune phenotype, are observed in both *A20*^myel-KO^ and *A20*^myel-KO^-*Padi4*^−/−^ mice (Fig. 3F, G). In accordance to the unaltered phenotype, PAD4 deficiency does not affect the amount of NETs in *A20*^myel-KO^ mice (Fig. 3H).

Finally, to more specifically address the potential role of A20 in neutrophils, we crossed A20 floxed mice with Mrp8-Cre^28^ mice to generate neutrophil-specific A20 knockout mice. Importantly, we found that in contrast to *A20*^myel-KO^ mice, neutrophil-specific A20 knockout mice do not develop splenomegaly and RA pathology, suggesting that A20 deficiency in neutrophils is not crucial for disease development (supplementary figure S7).

Together, these data demonstrate that protein citrullination and NET formation do not serve as pathogenic mechanisms driving RA pathology in myeloid A20-deficient mice.

## Discussion

The presence of increased protein citrullination and extracellular trap formation has been suggested as a possible explanation for the development of autoimmunity in individuals with defective A20 DUB function^12^. To investigate the impact of a non-functional A20-DUB mutation on autoimmunity development*,* we generated a knock-in mouse line carrying the C103R mutation. Unlike the commonly used substitution of the active site cysteine (C) with alanine (A), we chose to substitute the catalytic C with arginine (R) in this study. This decision was made based on previous findings indicating that the C-to-A mutation can significantly enhance the affinity of certain DUBs for ubiquitin, potentially leading to sequestration of cellular pools of monoubiquitin and ubiquitin chains^25^. As a result, the presence of such a mutation could render the A20-DUB into a high-affinity polyubiquitin-binding domain with unpredictable dominant negative physiological effects that are unrelated to the loss of enzymatic activity. To avoid this drawback, we evaluated the alternative option, substituting the active site cysteine with arginine (C103R), to inactivate A20’s DUB activity while also decreasing its affinity for ubiquitin, as previously suggested^25^. Our structural analysis strongly reinforces the justification for using this mutation by demonstrating that the C103R mutation in A20 effectively obstructs access to the A20 substrate binding site while leaving A20's structural integrity and active site unaltered. Direct evidence supporting this analysis is derived from the crystal structure of A20 after modification by iodoacetamide at position C103^27^, which illustrates how acetamidylation of C103 forms a structural adduct closely resembling the structural projection of an arginine mutation at C103 into the A20 DUB active site.

Our findings provide compelling evidence that loss of A20 DUB activity has minimal consequences, does not induce NETosis or give rise to SLE or RA pathology. Neither the previously published C103A mouse lines^22–24^ nor the C103R mouse line described in this study exhibit an inflammatory or autoimmune phenotype, underscoring the minor significance of A20's catalytic DUB function. These results align with recent studies highlighting that A20’s anti-inflammatory function is predominantly mediated through the ubiquitin-binding activity of zinc-fingers 4 and 7^10,26,29^.

In previous studies, we provided evidence that A20-deficient macrophages undergo necroptosis, mediated by RIPK1, RIPK3 and MLKL, leading to NLRP3 inflammasome activation and RA development in myeloid-specific A20 deficient mice^8–10^. Our current findings do not support a significant role for protein citrullination in the pathology of these mice, as demonstrated by the lack of rescue in RA pathology upon PAD4 deficiency. Furthermore, we found that in contrast to *A20*^myel-KO^ mice, neutrophil-specific A20 deletion in mice does not induce splenomegaly and RA pathology, suggesting that A20 deficiency in neutrophils is not crucial for disease development. Likewise, in DC-specific A20 deficient mice, PAD4 deficiency failed to ameliorate the SLE-like phenotype. Collectively, these experimental studies demonstrate that PAD4-dependent protein citrullination and NET formation do not act as a pathogenic mechanism in RA and SLE pathology in mice with A20 mutations, and likely not in individuals with A20 polymorphisms or mutations.

## Methods

### Mice

Conditional A20/*Tnfaip3* knockout mice, in which exons IV and V of the *Tnfaip3* gene are flanked by two LoxP sites, were generated as described before^30^. A20 floxed mice were crossed with LysM-Cre^31^, CD11c-Cre transgenic mice^32^ or Mrp8-Cre^28^ to generate a myeloid-specific (*A20*^myel-KO^), DC-specific (*A20*^DC-KO^) or neutrophil-specific A20 knockout mouse. A20^DUB^ knockin mice, that have a catalytic C103R mutation in the deubiquitinating OTU domain, were newly generated by CRISPR/Cas9 gene targeting. For this, Cas9 protein (500 ng/µl; VIB Protein Service Facility), together with a 123 bp single-stranded repair template (TGC < CGG (C103R): 5'-TTGCTTTGGGCTGCTTAACCTTGCTCCTCACAGCTCCTTCTGTCCTCAGGTGATGGAAACCGGCTCATGCATGCAGCTTGTCAGTACATGTGGGGTGTTCAGGATACTGACCTGGTCCTGAGG-3', iDT) and cr/tracrRNA complex (iDT; 100 ng/µl) with protospacer sequence 5′- ACTGACAAGCTGCATGCATG -3′) targeting the OTU domain of the murine *A20* gene (ENSMUSG00000019850), were electroporated into zygotes obtained from C57BL/6J mice. The embryos were transferred the same day to foster mothers through oviduct transfer. *Padi4* knockout mice were generated by electroporating Cas9 protein (iDT; 500 ng/µl) and cr/tracr RNA complexes (iDT; 100 ng/µl) with protospacer sequences 5’ GGCCTCCGGAATGGACTTTG 3’ and 5’ AGGATGGCGCCACGGCCGCC 3’ into zygotes obtained from C57BL/6 mice. This resulted in an allele with a 62 bp deletion in exon 5 (ENSMUSE00000279713) of the *Padi4* gene (ENSMUSG00000025330) at position chr4-: 140761632–140761693. This 62 bp deletion was predicted to cause amino acid sequence changes and a premature stop codon resulting in NMD. All experiments were performed on male and female mice of C57BL/6 genetic background. Mice were housed in individually ventilated cages at the VIB Center for Inflammation Research in a conventional animal facility. All experimental protocols on mice were performed according to institutional, national and European animal regulations.

### Cell culture studies

Bone marrow was flushed from mouse femurs and tibia with ice-cold sterile RPMI medium, and cultured in Roswell Park Memorial Institute 1640 medium supplemented with 40 ng/mL recombinant mouse macrophage colony-stimulating factor (M-CSF) (VIB Protein Core), 10% fetal calf serum, L-glutamine (200 mM) and 1% penicillin/streptomycin at 37 °C and 5% CO2. Fresh M-CSF was added on day 3 and medium was refreshed on day 5. On day 7 cells were seeded at 1 × 10^6^ cells in adherent 6-well plates and stimulated with 20 ng/mL LPS or 20 ng/mL mTNF for the indicated time points. Immortalized A20-deficient mouse embryonic fibroblasts (MEFs) ^33^ were cultured in Dulbecco’s modified Eagle’s medium supplemented with 10% fetal calf serum, L-glutamine (200 mM), sodium pyruvate (400 mM) and 1% penicillin/streptomycin at 37 °C and 5% CO2. Cells were stably reconstituted by lentiviral transduction with either wild type (WT) A20 or one of two deubiquitinase (DUB) inactive versions of A20 (A20 C103A or C103R). Entry vectors coding for each A20 DUB mutant were generated by QuikChange site-directed mutagenesis (Agilent) according to the manufacturer’s instructions. For this, following primers were used: TGC > GCC (C103A); 5’-CGA ACG GTG ACG GCA ATG CCC TCA TGC ATG CCA CTT C-3’ (Fw) and 5’-GAA GTG GCA TGC ATG AGG GCA TTG CCG TCA CCG TTC G-3’ (Rev) and TGC > AGG (C103R); 5’-GAA CGG TGA CGG CAA TAG GCT CAT GCA TGC CAC TTC-3’ (Fw) and 5’-GAA GTG GCA TGC ATG AGC CTA TTG CCG TCA CCG TTC-3’ (Rev). MEFs were seeded at 200,000 cells per well in triplicate in adherent 6-well plates and treated with 1 µg/mL doxycycline for 24 h prior to stimulation. Cells were stimulated with 10 or 20 ng/mL mTNF for the indicated time points.

### Isolation of neutrophils from bone marrow

Bone marrow cells were flushed from mouse tibia and fibula with ice-cold sterile RPMI medium over a 70 µm cell strainer. Following centrifugation (1240 rpm for 4 min at room temperature), red blood cells were lysed by incubation with NaCl solution (0.2% NaCl:1.2% NaCl, 4:11). Cells were spun down (1240 rpm for 5 min at room temperature), resuspended in PBS and slowly added on top of a 62% Percoll (cells:Percoll, 1:1) (Percoll TM: Cytiva, cat nr. 17089101). Density gradient centrifugation was performed (2500 rpm for 28 min at room temperature), and the lower neutrophil containing fraction was washed with PBS and spun down (1240 rpm for 4 min at room temperature). Pellets were resuspended in RPMI**.**

### FACS

Spleens were cut into small pieces and digested at 37 °C for 30’ in RPMI containing 1% fetal bovine serum, 20 µg/mL Liberase TM (Roche) and 10 U/mL DNAse I (Roche). Following digestion, cells were passed through a 70 µm filter. Osmotic lysis buffer was added on ice for 3 min to remove erythrocytes. This digest was resuspended and 5% of the cells were used for further processing. Counting beads (eBioscience cat. 01-1234-42) were added and the cells were stained with fluorescently labeled antibodies and Fc block during 30 min at 4 °C. Antibodies used : Anti-mouse CD11b monoclonal antibody (rat, clone M1/70, BD Biosciences, 563553), Anti-mouse Ly-6G monoclonal antibody (rat, clone 1A8, BD Biosciences, 612921), Anti-mouse CD26 monoclonal antibody (rat, clone H194-112, BD Biosciences, 741729), Anti-mouse Ly-6C monoclonal antibody (rat, clone HK1.4, eBioscience, 48-5932-82), Anti-mouse CD45 monoclonal antibody (rat, clone 30-F11, BioLegend, 103138), Anti-mouse XCR1 monoclonal antibody (mouse, clone ZET, BioLegend, 148220), Anti-mouse CD64 monoclonal antibody (mouse, clone X54-5/7.1, BioLegend, 139311), Anti-mouse F4/80 monoclonal antibody (rat, clone BM8, BioLegend, 123141), Anti-mouse MerTK monoclonal antibody (rat, clone 2B10C42, BioLegend, 151504), Anti-mouse I-A/I-E monoclonal antibody (rat, clone M5/114.15.2, BioLegend, 107626), Anti-mouse CD88 monoclonal antibody (rat, clone 20/70, BioLegend, 135806), Anti-mouse CD11c monoclonal antibody (Armenian hamster, clone N418, eBioscience, 61-0114-82), Anti-mouse CD19 monoclonal antibody (rat, clone 1D3), eBioscience, 15-0193-83), Anti-mouse CD3 monoclonal antibody (Armenian hamster, clone 145-2C11, eBioscience, 15-0031-83), Anti-mouse Ter119 monoclonal antibody (rat, clone TER119, eBioscience, 15-5921-82), Anti-mouse NK1.1 monoclonal antibody (mouse, clone PK136, BioLegend, 108716), Anti-mouse CD172a monoclonal antibody (rat, clone P84, BioLegend, 144008), Anti-mouse 120G8 monoclonal antibody (rat, clone 120-G8, produced in house), Fixable Viability Dye eFluor780, eBioscience, 65-0865-14), Anti-mouse TCR delta monoclonal antibody (Armenian hamster, clone GL3, BD Biosciences, 553177), Anti-mouse CD8a monoclonal antibody (rat, clone 53–6.7, eBioscience, 45-0081-82), Anti-mouse CD62L monoclonal antibody (rat, clone MEL-14, eBioscience, 11-0621-85), Anti-mouse CD44 monoclonal antibody (rat, clone IM7, BioLegend, 103049), Anti-mouse CD4 monoclonal antibody (rat, clone RM4-5, eBioscience, 170042-83), Anti-mouse CD3 monoclonal antibody (Armenian hamster, clone 145-2C11, eBioscience, 12-0031-82), Anti-mouse CD45R monoclonal antibody (rat, clone RA3-6B2, BD Biosciences, 563708), Anti-mouse CD19 monoclonal antibody (rat, clone 1D3, BD Biosciences, 563333), Anti-mouse GL7 monoclonal antibody (rat, clone GL7, BioLegend, 144606), Anti-mouse CD95 monoclonal antibody (hamster, clone Jo2, BD Biosciences, 557653). All samples were measured on a BD LSRFortessa. Downstream analysis was performed in Flowjo (BD).

### Clinical scoring for arthritis development

Mice were randomly scored in a blinded fashion for development of peripheral arthritis. The severity of arthritis was assessed using a visual scoring system.

### Histology

Paraffin sections of paws were made at 7 µm thickness and stained with haematoxylin and eosin for evaluation of inflammation and bone erosion. Histological scores were based on evaluation of four parameters, at the Achilles tendon (infiltrate), the synovio-entheseal complex (exudate), the talus-tibia-calcaneus (exudate), and calcaneal erosion, each ranging from 0 (normal) to 3 (severely inflamed). Paraffin sections of kidney were made at 3 µm thickness, and of colon, spleen and liver at 5 µm thickness and stained with haematoxylin and eosin or periodic acid-Schiff (PAS).

### Antinuclear antibodies

Specific ANA were detected by line immunoassay (INNO-LIA ANA Update, Innogenetics NV). The nylon strips were incubated with serum at a 1:200 dilution. Following washing, a 1:2500 dilution of an alkaline phosphatase-conjugated anti-mouse IgG was added (Chemicon). After washing, the reaction was revealed with the chromogen 5-bromo-4-chloro-3-indolyl phosphate, producing a dark brown color in proportion to the amount of specific autoantibody in the test sample. Sulfuric acid was added to stop the color development. The assay contains the following recombinant and natural antigens: SmB, SmD, RNP-A, RNP-C, RNP-70 k, Ro52/SSA, Ro60/SSA, La/SSB, CenpB, Topo-I/Scl70, Jo-1, ribosomal P, and histones. Alternatively, mouse anti-dsDNA IgG or IgA were quantified on ELISA (Alpha Diagnostic Intl., cat. 5120) according to the manufacturers’ instructions. For the measurement of anti-cardiolipin antibodies, microplates were coated with 50 µg/mL cardiolipin from bovine heart (Sigma, cat. C1649) in 100% ethanol. Following overnight incubation at room temperature, plates were blocked with 1% bovine serum albumin in PBS. After serum incubation for 2 h, HRP-labeled goat anti-mouse IgG or IgA (Southern Biotech cat. 1030-05 and 1040-05 resp.) was added for 1 h and detected by TMB (eBioscience cat 00-4201-56). The absorbance for each sample was measured at 450 and 650 nm.

### Cytokine detection

Cytokine levels in culture medium were determined by magnetic bead-based multiplex assay using Luminex technology (Bio-Rad) according to the manufacturers’ instructions. Alternatively, mouse IL-6 (Invitrogen cat. 88-7064) and TNF (Invitrogen cat. 88-7324) were measured by ELISA, according to the manufacturer’s instructions. B-cell activating factor (BAFF) was quantified on ELISA (R&D cat DY2106-05) according the manufacturer’s instructions. Neutrophil extracellular traps (NETs) were detected by ELISA (Merck cat. 11774425001) according to the manufacturers’ instructions.

### Western blotting

Cells were directly lysed in 2 × Laemmli or first lysed in RIPA lysis buffer (50 mM Tris–HCl pH 7.6, 1 mM EDTA, 150 mM NaCl, 1% NP40, 0.5% sodiumdeoxycholate, 0.1% SDS) buffer, followed by denaturation in 1 × Laemmli buffer (5% β-mercaptoethanol, 100 mM Tris–HCl (pH 6.8), 10% glycerol, 2% SDS and bromophenol blue) and boiled for 5 min. Lysates were separated by SDS polyacrylamide gel electrophoresis, transferred to nitrocellulose membranes with a semi-dry blot system (Invitrogen), and immunoblotted with anti-A20 (Santa Cruz, sc-166692), anti-IκBα (Santa Cruz Biotechnology, Inc., sc-371), anti-phospho-IκBα (Cell Signaling, CST9246), anti-p38 (cell signaling, CST9212), anti-phospho-p38 (cell signaling, CST9215), anti-SAPK/JNK (cell signaling, CST9252), anti-phospho-SAPK/JNK (cell signaling, CST4668), anti-PAD4 (Abcam, Ab214810), and anti-β-actin (Santa Cruz Biotechnology, Inc., sc-47778) antibodies.

### Immunofluorescence

The kidneys were freshly frozen in OCT (Sakura, 4583) and stored at −80 °C. Cryosections of 10 to 20 µm thickness were fixed for 2 to 10’ in 2% PFA at room temperature. For immunoglobulin detection, sections were blocked for 60’ with 1% goat and 1% rat serum. Following washing, anti-mouse IgA monoclonal antibody (rat, clone C10-3, FITC conjugated, BD Biosciences, #559354) and DAPI (ThermoFisher, D21490) were added for 2 h. For neutrophil extracellular trap (NET) imaging, tissues were blocked in 2% BSA (Sigma-Aldrich) and 3% Donkey serum for 60’ at room temperature. Tissues were stained overnight at 4 °C with antibodies against myeloperoxidase (R&D, AF3667-SP) and cit-H3 (Abcam, ab5103), and then washed three times for 30’ in total. Tissues were then stained with secondary antibodies against goat or rabbit for 1 h at room temperature, and then stained with DAPI (ThermoFisher, D21490) for 15 min. Slides were mounted with polyvinyl alcohol (Sigma, 10981), and imaged on a laser scanning microscope (Zeiss LSM-780, immunoglobulin detection) or a slide scanner (Zeiss Axioscan, NET imaging). For the immunoglobulin detection, quantification of fluorescent intensity was carried out in ImageJ. The Z stacks were compressed at maximal intensity, the glomerular area were manually marked and the mean fluorescent intensity was measured per glomerular area for each fluorophore. The mean of 3 to 5 representative glomeruli per mouse was plotted.

### Quantitative PCR

Whole spleen was homogenized with a TissueLyser (Qiagen) and further processed for RNA extraction using TRIzol Reagent (Invitrogen cat. 10296-010) according to the manufacturer’s instructions. RNA content was measured on a NanoDrop Spectrophotometer (Thermo Scientific) and 1 µg RNA was transferred for cDNA conversion using the sensiFAST cDNA synthesis kit (Bioline cat. 65054). cDNA of interest was amplified by 30 cycles of PCR with sensiFAST SYBR No-ROX kit (Bioline cat. 98050) on a LightCycler 480 system (Roche). The following primers were used: target genes Isg15 (GGTGTCCGTGACTAACTCCAT, TGGAAAGGGTAAGACCGTCCT), Isg20 (GAACATCCAGAACAACTGGCG, GTAGAGCTCCATTGTGGCCCT), Rsad2 (GGTGCCTGAATCTAACCAGAAG, CCACGCCAACATCCAGAATA) and reference genes TBP (TCTACCGTGAATCTTGGCTGTAAA, TTCTCATGATGACTGCAGCAAA), HPRT (TCCTCCTCAGACCGCTTT, CCTGGTTCATCATCGCTAATC). Analysis was carried out in qbase + (Biogazelle) and Calibrated Normalized Relative Quantities (CNRQs) values were exported.

### Structural modelling

The crystal structure of human A20 (pdb code 3DKB^34^) was visualized in PyMOL version 2.5.4 and computationally mutated at C103 to an arginine residue by selecting the most frequent rotamer for the arginine side chain. Comparison of this structure carrying a C103R mutation by superposition to human A20 DUB modified by iodocetamide at C103 leading to acetamidylated C103 (PDB code 53VB^27^) was carried out in PyMOL version 2.5.4 using the native protein structure alignment routines.

### Statistics and reproducibility

GraphPad Prism V8 software was used for statistical analysis. Results are expressed as the mean ± SEM or mean ± SD, as indicated in figure legend. Statistical significance between experimental groups was assessed using a nonparametric Mann–Whitney U-statistical test. Statistical significance between multiple groups was assessed using either one- or two-way ANOVA with Tukey or Sidak correction for multiple comparison. Comparison of two or more groups over time was analyzed as longitudinal data (repeated measurements over time) using the residual maximum likelihood (REML) as implemented in Genstat v.19.

### Ethics statement

All animal experiments were carried out in accordance with relevant guidelines and regulations, as described by the ARRIVE guidelines. The Ethical committee of The Faculty of Sciences of Ghent University approved all the animal experiments.

### Supplementary Information

Supplementary Information 1.

Supplementary Information 2.

## Data Availability

All data supporting the findings of this study are available within the paper and its Supplementary Information.
