# Supplementary material for: Protein citrullination and NET formation do not contribute to the pathology of A20/TNFAIP3 mutant mice
Source: Sci Rep. 2023 Oct 21;13:17992. doi: 10.1038/s41598-023-45324-8 (PMC10590390; doi:10.1038/s41598-023-45324-8)
Supplement: Supplementary file 1 — Supplementary Information 1. [file 41598_2023_45324_MOESM1_ESM.pdf]

Protein citrullination and NET formation do not contribute to the pathology of A20/TNFAIP3 mutant mice

Karel F. A. Van Damme[1,2,+], Pieter Hertens[1,3,+], Arne Martens[1,3], Elisabeth Gilis[1,4], Dario Priem[1,3], Inge Bruggeman[1,3], Amelie Fossoul[1,3], Jozefien Declercq[1,2], Helena Aegerter[1,2], Andy Wullaert[1,3,5], Tino Hochepied[1,3], Esther Hoste[1,3], Lieselotte Vande Walle[2], Mohamed Lamkanfi[2], Savvas N. Savvides[1,6], Dirk Elewaut[1,4], Bart N. Lambrecht[1,2,7,*] and Geert van Loo[1,3,*]

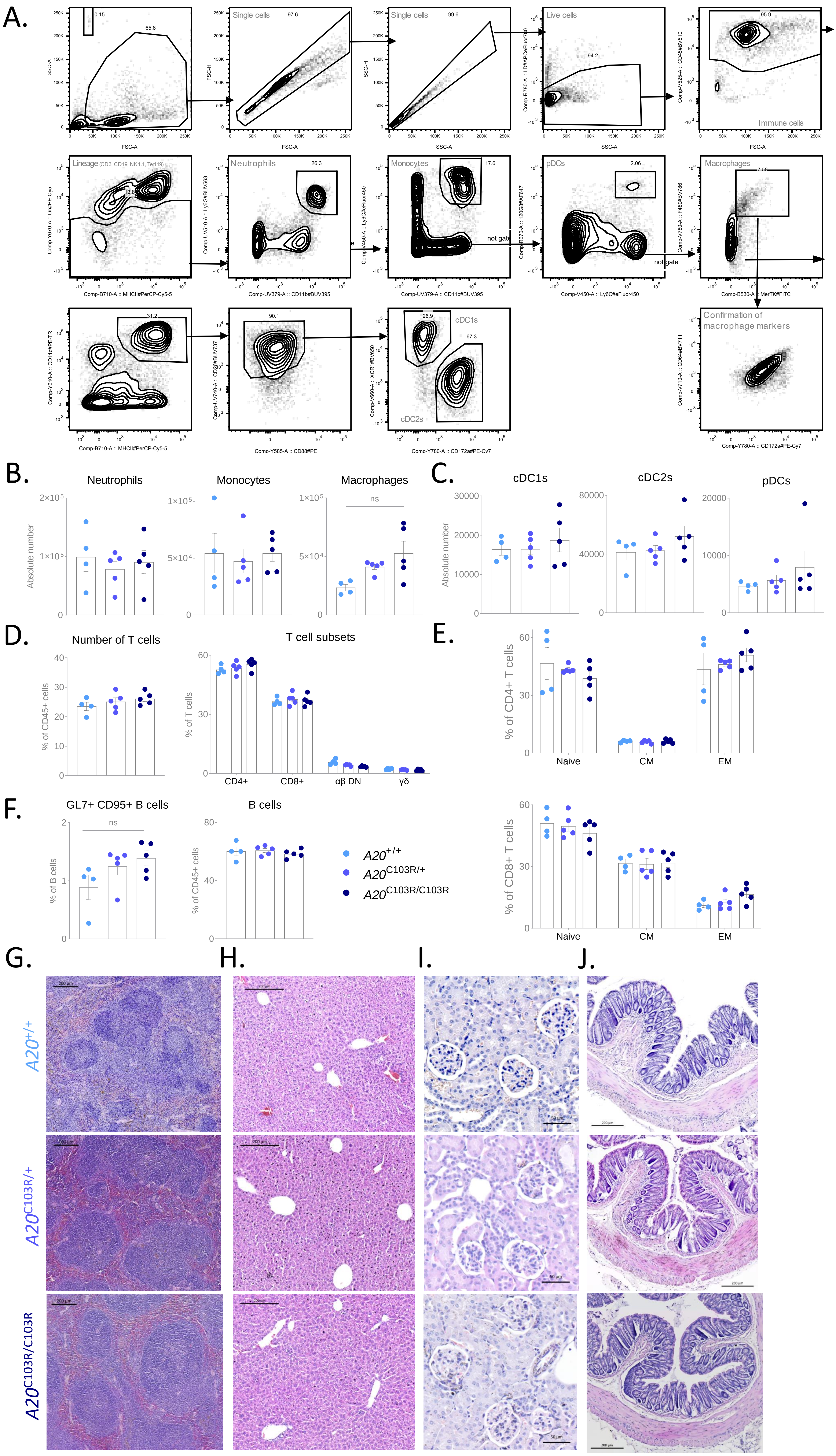

Supplementary Figure S1. A20 DUB mutation does not induce autoimmune pathology. (A) Gating of myeloid and dendritic cells in spleen (shown: wild-type mouse). (B-E) Numbers of myeloid cells (B), DCs (C), T cells (D-E) and B cells (F) in 25-30 week old wild-type (A20$^{+/+}$), A20$^{C103R/+}$ and A20$^{C103R/C103R}$ mice. Each dot represents one mouse. Data are expressed as mean ± s.e.m. (G-J) Representative H&E stained sections of spleen (G), liver (H), kidney (I) and distal colon (J), of wild-type, A20$^{C103R/+}$ and A20$^{C103R/C103R}$ mice above the age of 40 weeks.

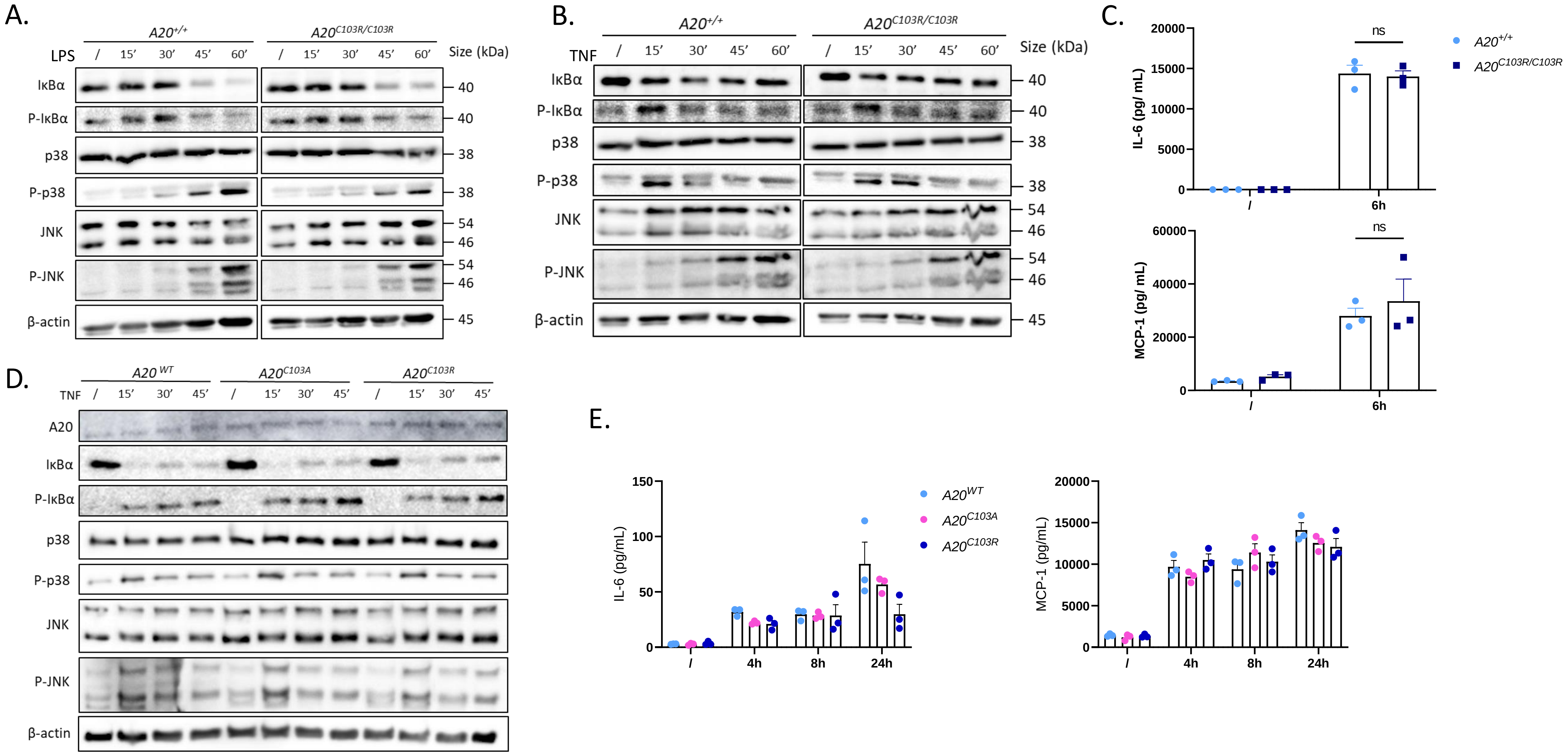

Supplementary Figure S2. A20 DUB mutation does not affect NF-kB activation. (A-B) Immunoblot analysis of whole-cell lysates from A20$^{+/+}$ and A20$^{C103R/C103R}$ BMDMs stimulated with LPS (20 ng/ml) (A) or TNF (20 ng/ml) (B), as indicated. β-actin is shown as a loading control. (C) Levels of IL-6 and MCP-1 in supernatant of BMDMs isolated from control A20$^{+/+}$ (n = 3) and A20$^{C103R/C103R}$ (n = 3) mice, either or not stimulated with LPS for 6 h. Data are expressed as mean ± s.e.m. (D) A20$^{-/-}$ MEFs were lentivirally reconstituted with doxycycline-inducible wild-type A20 (A20^{WT}), A20^{C103A} or A20^{C103R} constructs. Cells were treated with 1 µg/mL doxycycline for 24 h prior to stimulation with 20 ng/mL TNF. Immunoblot analysis of whole-cell lysates from MEFs. β-actin is shown as a loading control. (E) Levels of IL-6, and MCP-1 in supernatant of A20^{WT}, A20^{C103R} and A20^{C103A} MEFs stimulated with 10 ng/mL TNF.

Supl. Fig. S3

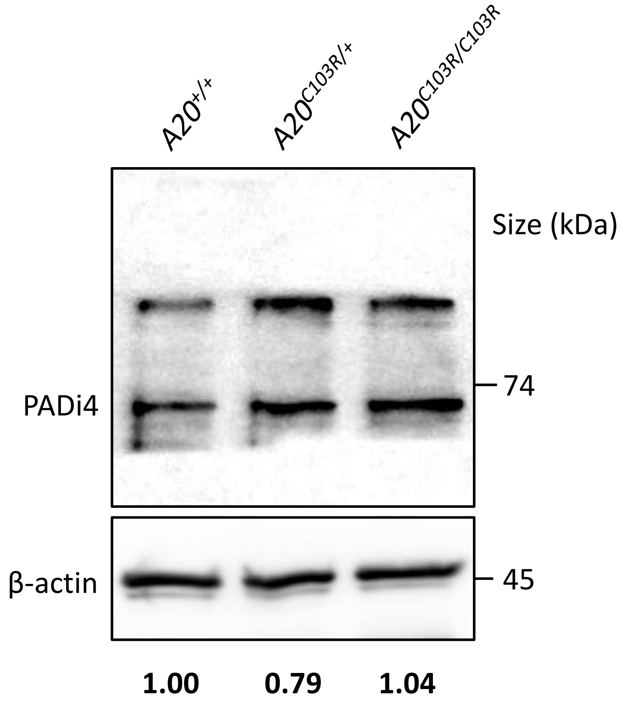

Supplementary Figure S3. A20 DUB mutation does not induce enhanced PAD4 expression. Immunoblot analysis of whole-cell lysates from neutrophils isolated from A20$^{+/+}$, A20$^{C103R/+}$ and A20$^{C103R/C103R}$ mice. β-actin is shown as a loading control. The quantitative measurement of PAD4 band intensity (normalized to actin) is shown below. Data are representative of 3 independent experiments (using different mice).

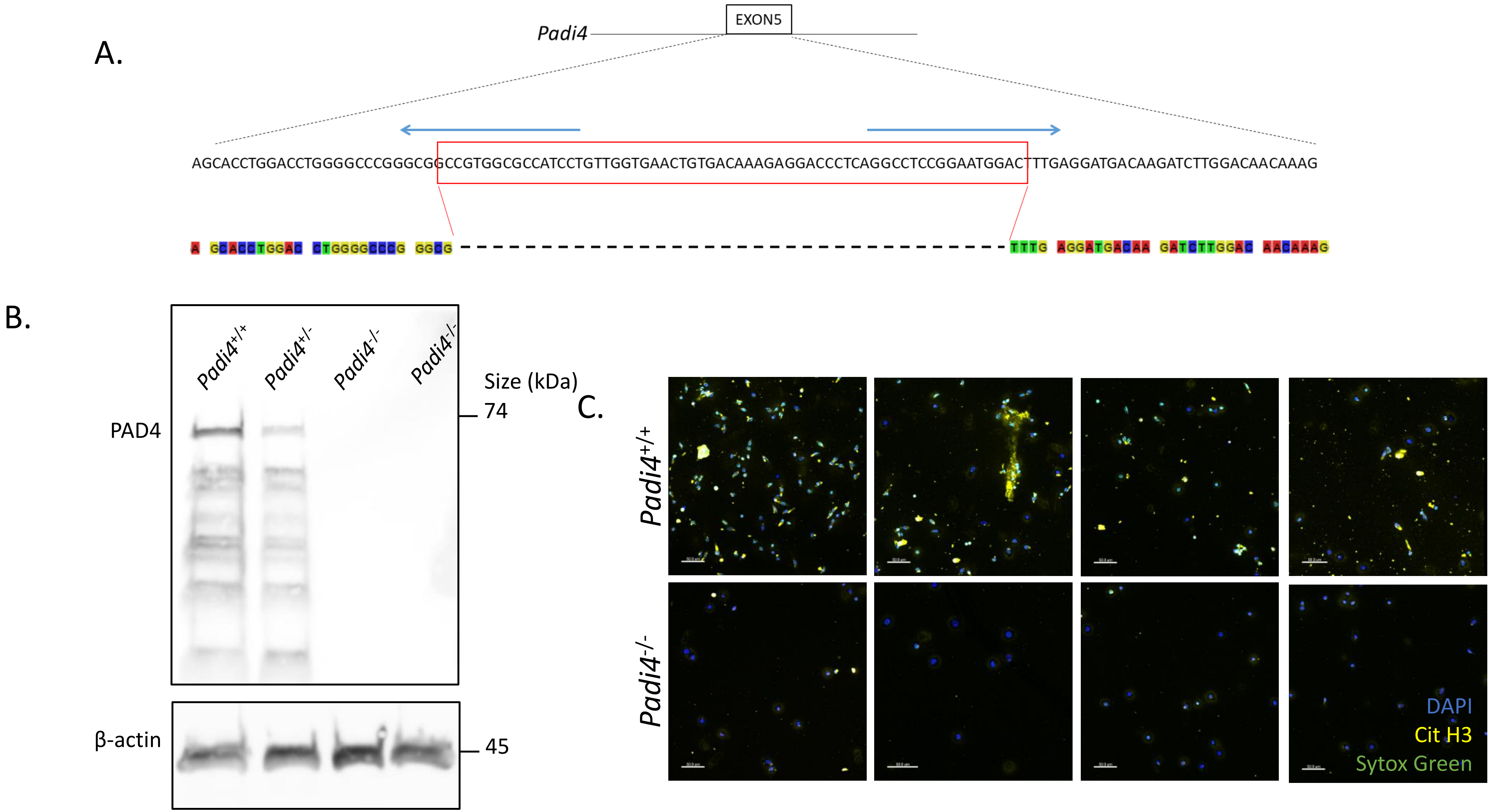

Supplementary Figure S4. *Padi4* targeting strategy. (A) Exon5 of the *Padi4* gene was targeted with 2 gRNAs with protospacer sequences indicated with blue arrows. The 62 bp deletion in exon 5 resulting from electroporation with Cas9 RNP complexes containing both gRNAs is indicated with a red box. Sequencing results show the 62 bp deletion in exon 5. (B) Immunoblot analysis of whole-cell lysates from neutrophils isolated from *Padi4*$^{+/+}$, *Padi4*$^{+/-}$ and *Padi4*$^{-/-}$ mice. β-actin is shown as a loading control. (C) Neutrophils isolated from *Padi4*$^{+/+}$ and *Padi4*$^{-/-}$ mice were stimulated for 4 hours with 100ng/ml PMA and analysed for the presence of NETs by immunostaining of citH3. Scalebar 25 µm.

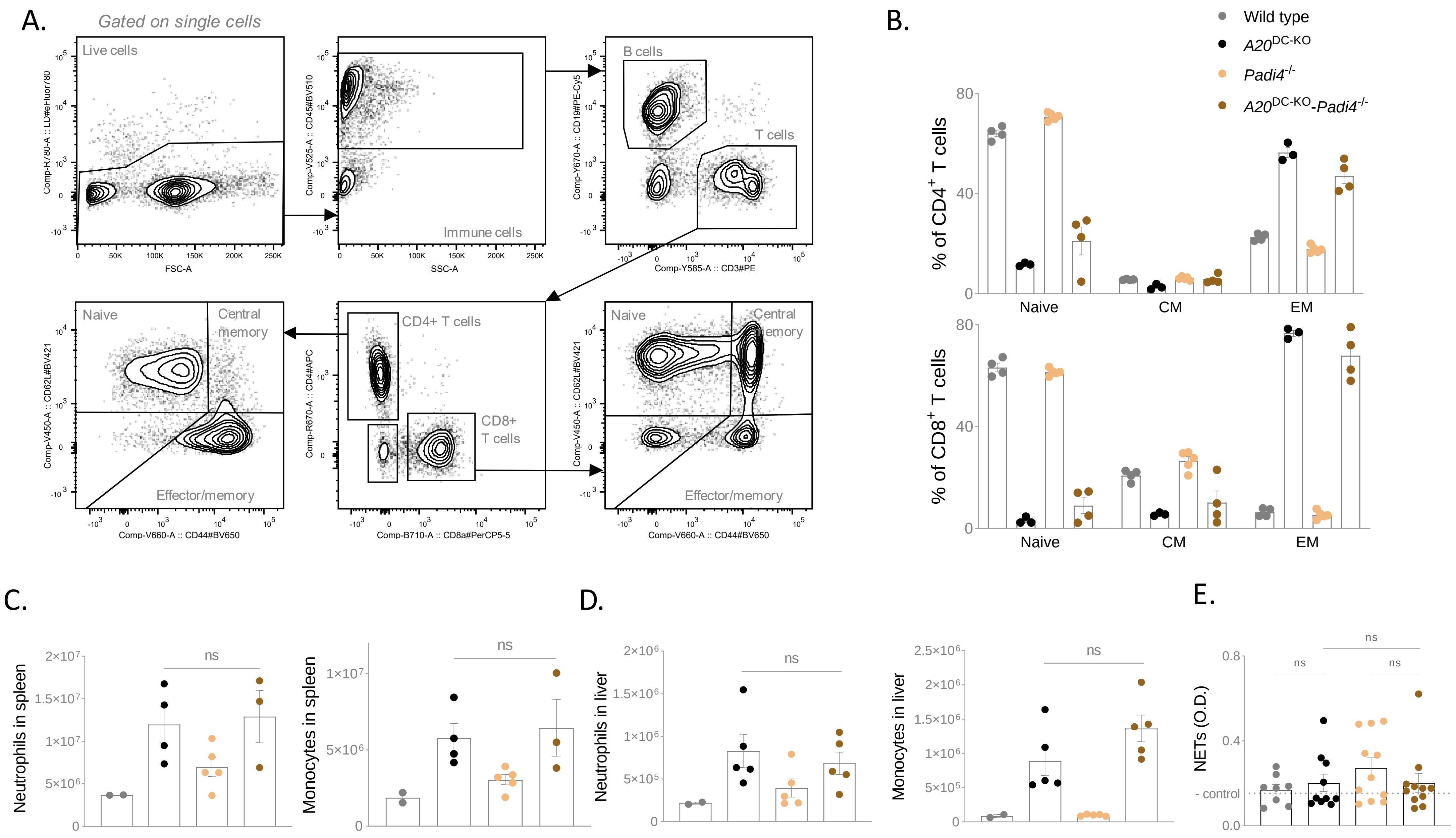

Supplementary Figure S5. Padi4-deficiency does not restore spleen and liver immune cell composition in A20^{DC-KO} mice. (A) Gating strategy on flow cytometry to discriminate T cell subsets. (B) T cell activation, expressed as a percentage of CD62L+CD44- or CD44+CD62L- cells of total CD4+ or CD8+ T cells. (C-D) Number of neutrophils and monocytes in spleen (C) and liver (D). (E) Neutrophil Extracellular Traps (NETs) in serum. Data are expressed as mean ± s.e.m.

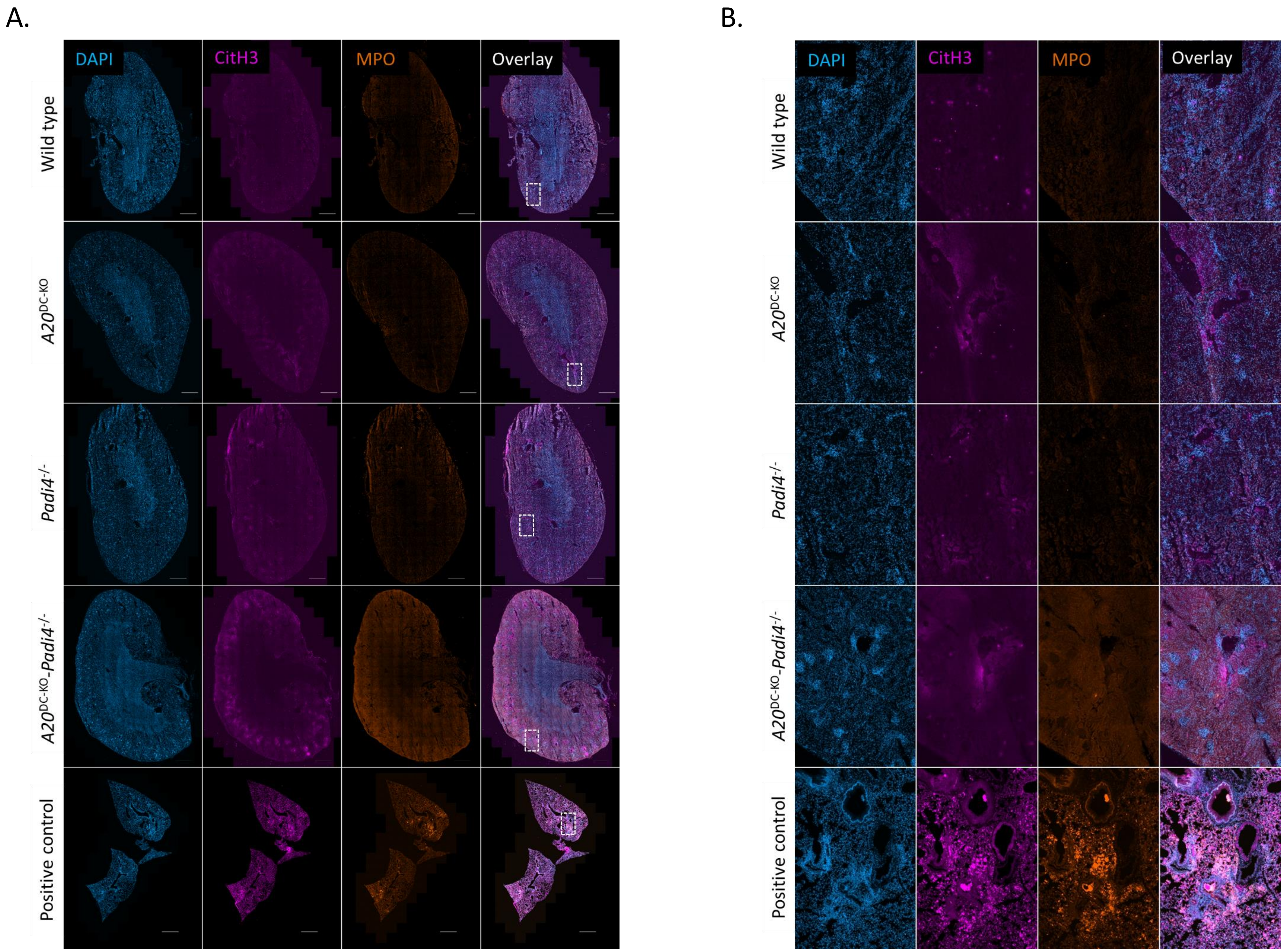

Supplementary Figure S6. A20-deficiency in dendritic cells does not lead to neutrophil extracellular traps in the kidney. (A) Overview of immunofluorescent images of neutrophil extracellular traps in the kidney, for each genotype. (B) Region of interest for each genotype. Blue: DAPI; Purple: citrullinated histone H3 (CitH3); Orange: myeloperoxidase (MPO). Pictures are representative for 4-5 biologically independent mice for each genotype. Scalebar, 1000 μm.

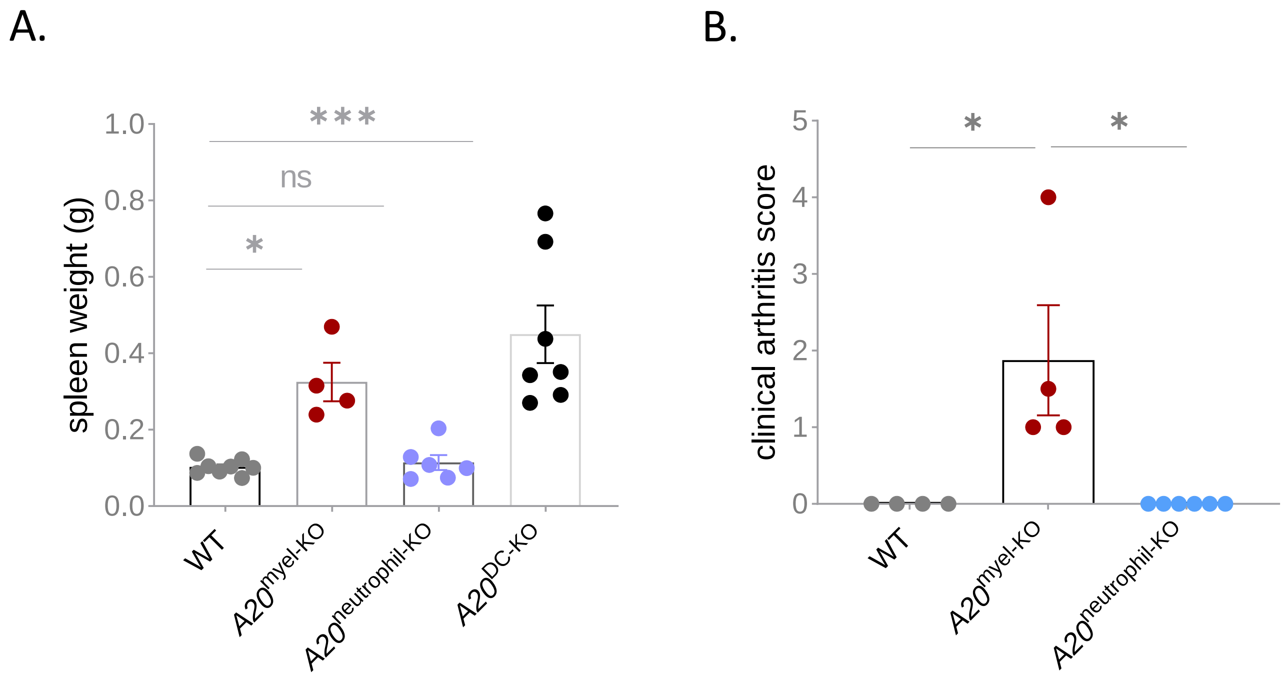

Supplementary Figure S7. Neutrophil-specific A20 knockout mice do not develop pathology. (A) Spleen weight of 25-30 week old wild-type, *A20*$^{\text{myel-KO}}$, *A20*$^{\text{DC-KO}}$ and *A20*$^{\text{neutrophil-KO}}$ mice. Each dot represents one mouse. Data are expressed as mean ± s.e.m. (B) Clinical arthritis scores of the ankles of wild-type, *A20*$^{\text{myel-KO}}$, *A20*$^{\text{neutrophil-KO}}$ mice at the age of 25-30 weeks. Data are expressed as mean ± s.e.m.
