# Supplementary material for: Protein citrullination and NET formation do not contribute to the pathology of A20/TNFAIP3 mutant mice
Source: Sci Rep. 2023 Oct 21;13:17992. doi: 10.1038/s41598-023-45324-8 (PMC10590390; doi:10.1038/s41598-023-45324-8)
Supplement: Supplementary file 2 — Supplementary Information 2. [file 41598_2023_45324_MOESM2_ESM.pptx]

## Slide 1
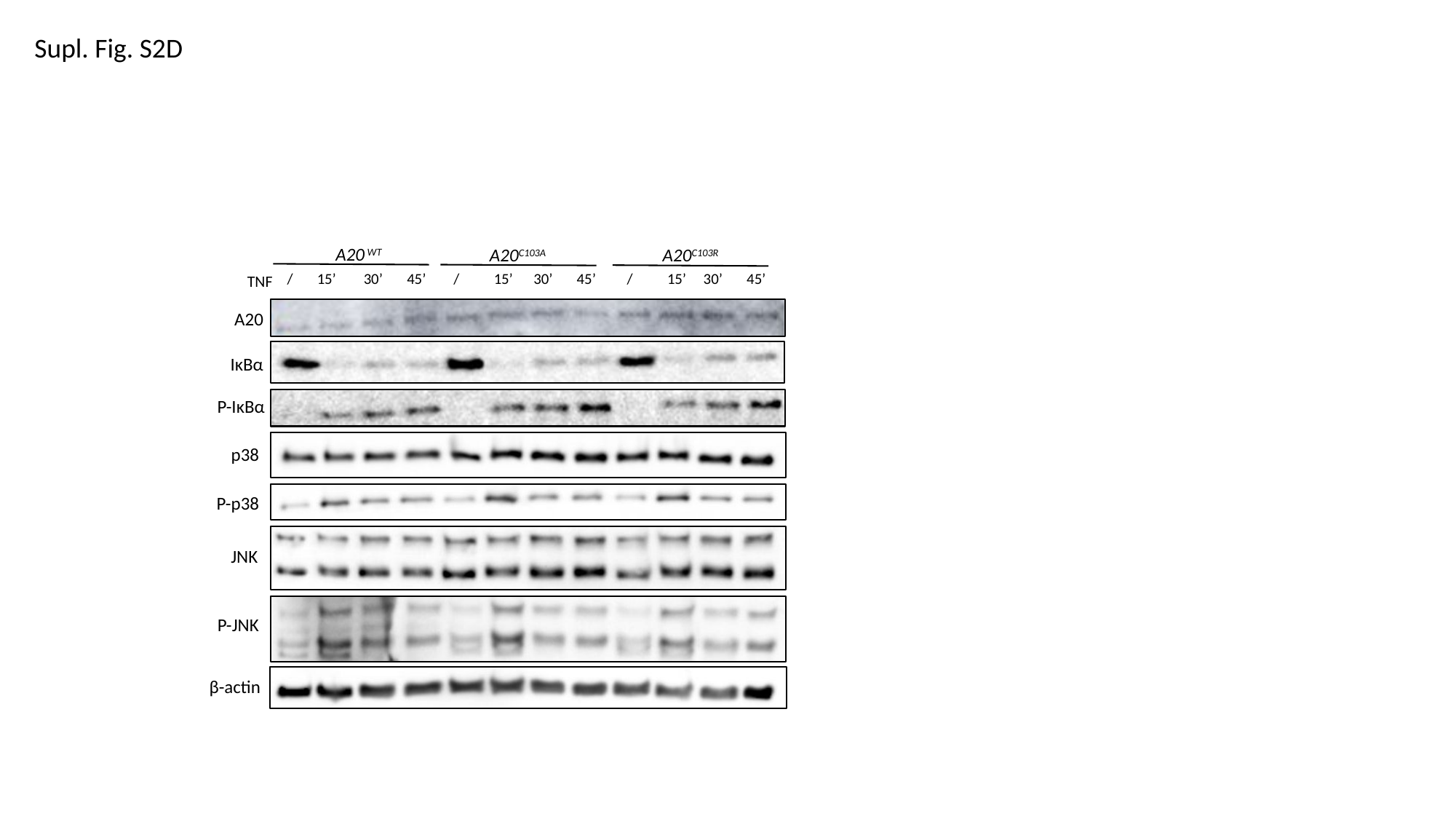

Supl. Fig. S2D
A20 WT
A20C103A
A20C103R
/ 15’ 30’ 45’ / 15’ 30’ 45’ / 15’ 30’ 45’
TNF
A20
IκBα
P-IκBα
p38
P-p38
JNK
P-JNK
β-actin

## Slide 2
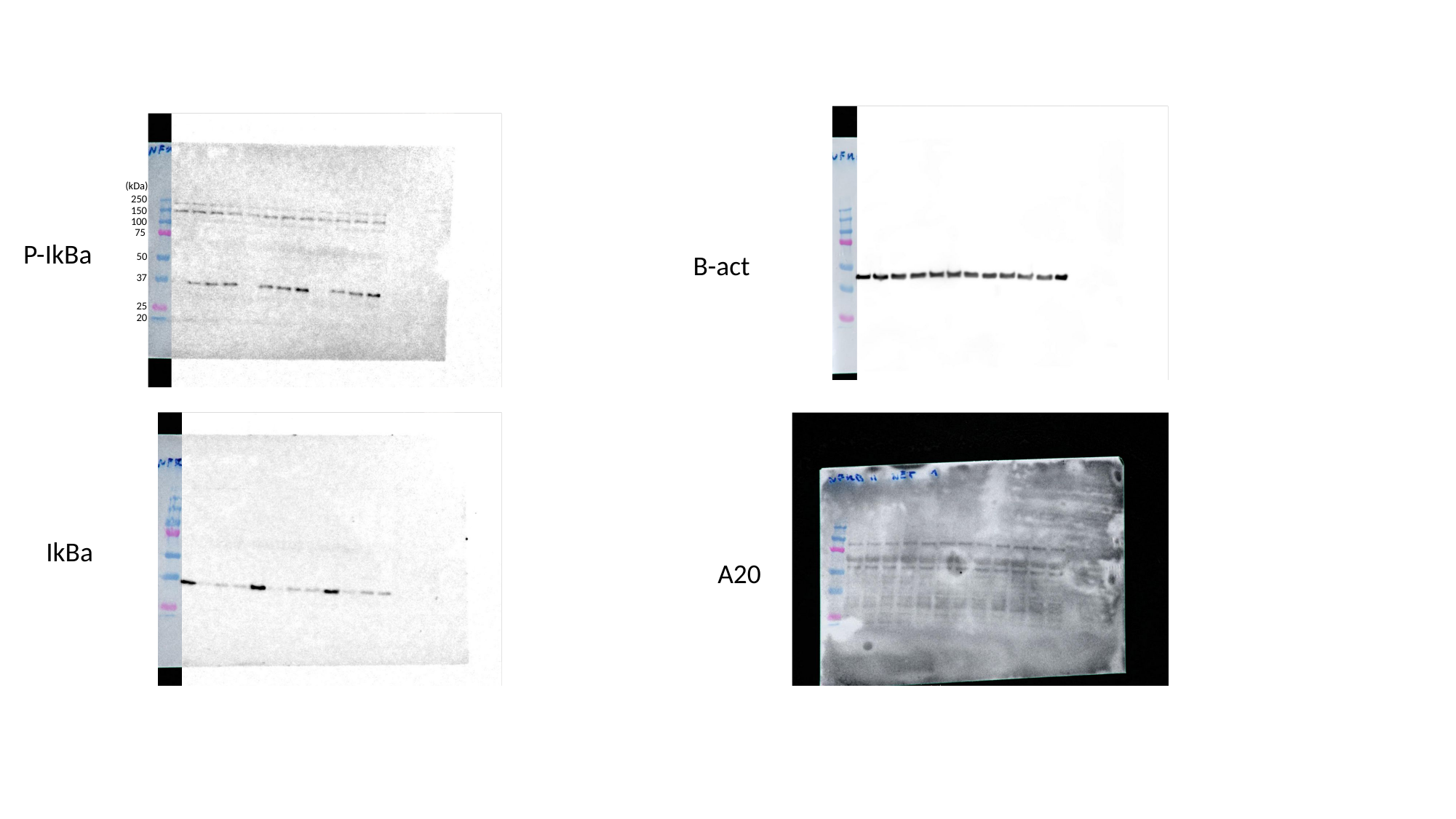

(kDa)
250
150
100
75
P-IkBa
B-act
50
37
25
20
IkBa
A20

## Slide 3
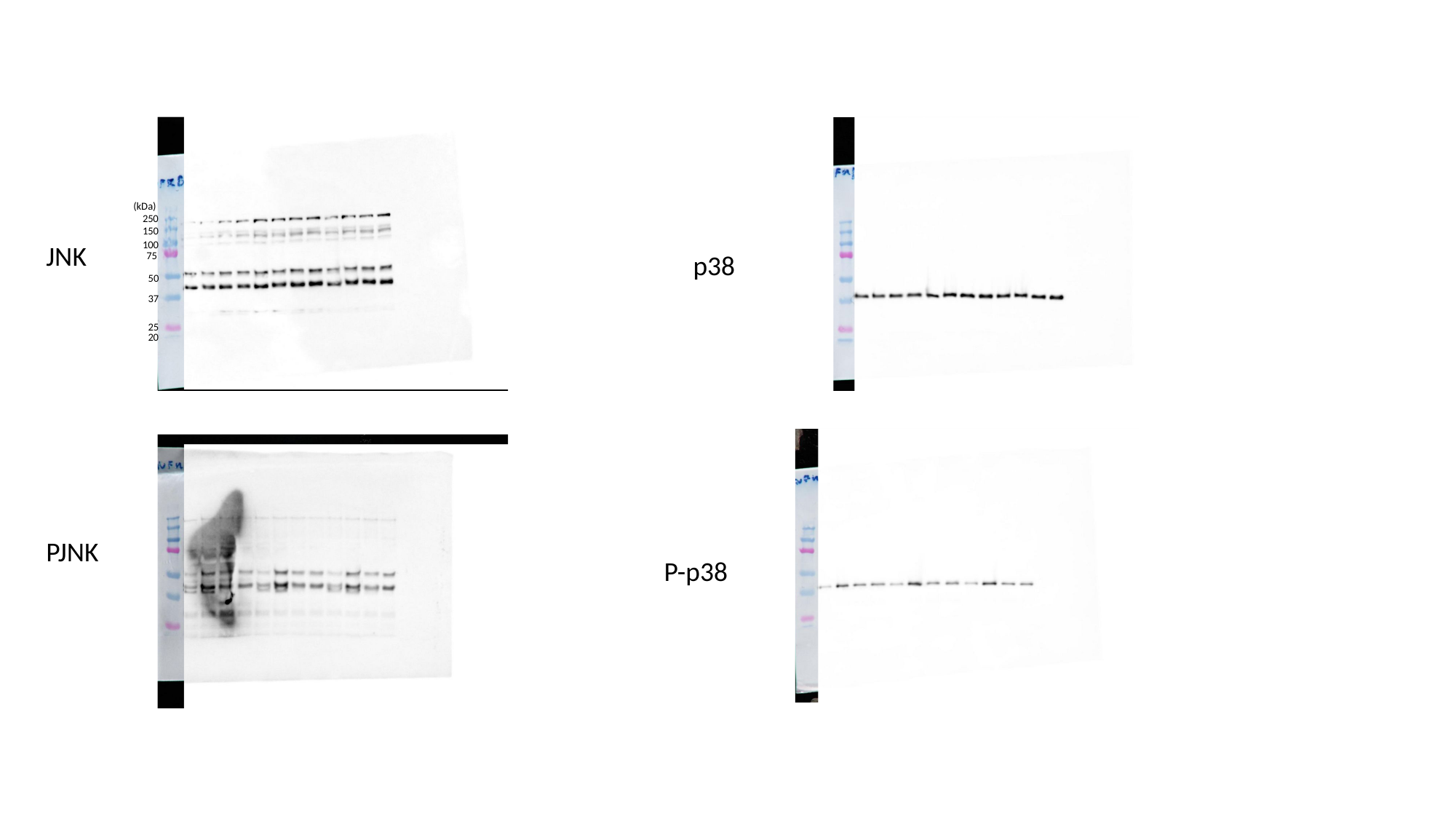

(kDa)
250
150
100
JNK
p38
75
50
37
25
20
PJNK
P-p38

## Slide 4
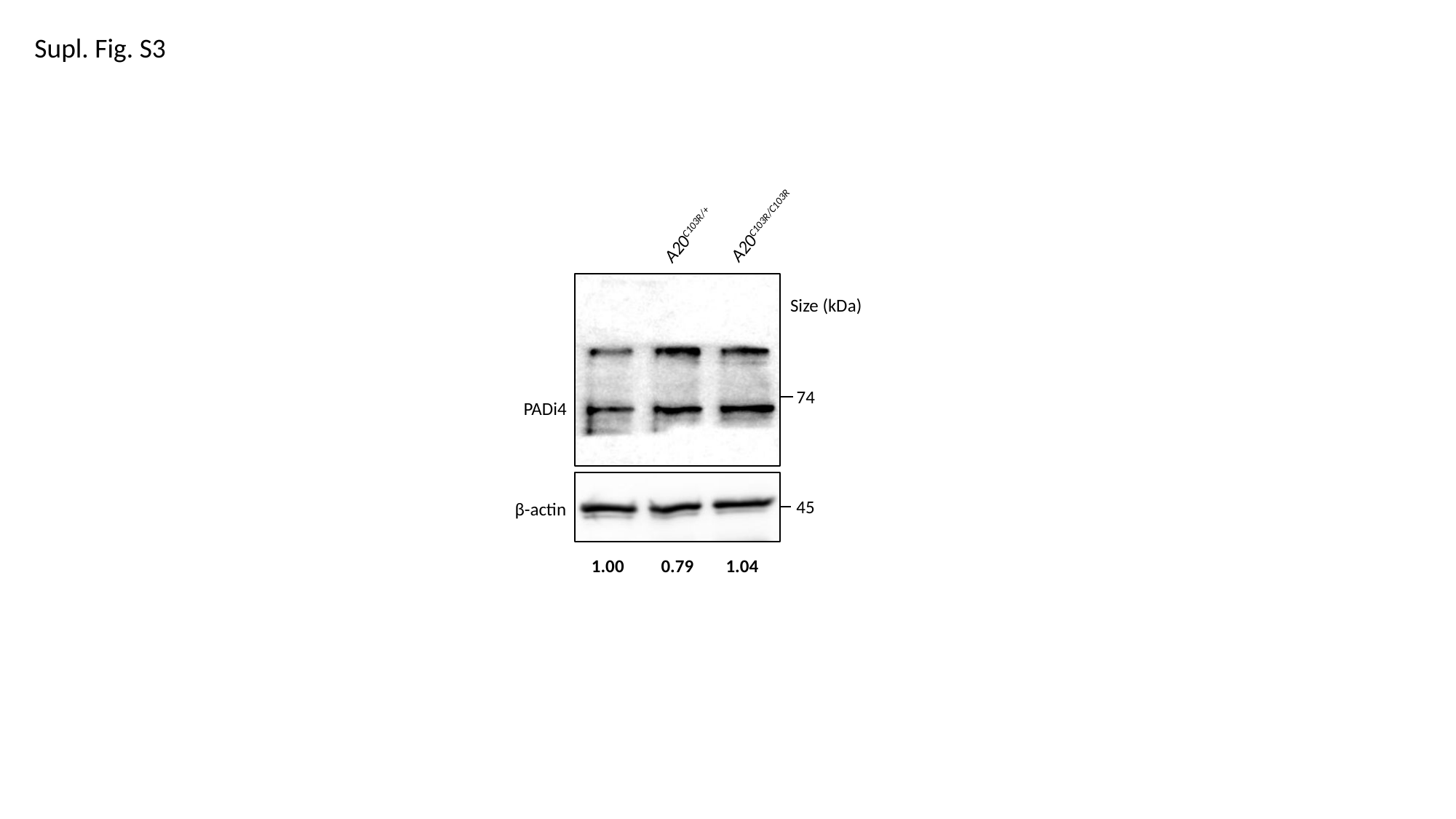

Supl. Fig. S3
A20C103R/C103R
A20C103R/+
Size (kDa)
74
PADi4
45
β-actin
1.00
0.79
1.04

## Slide 5
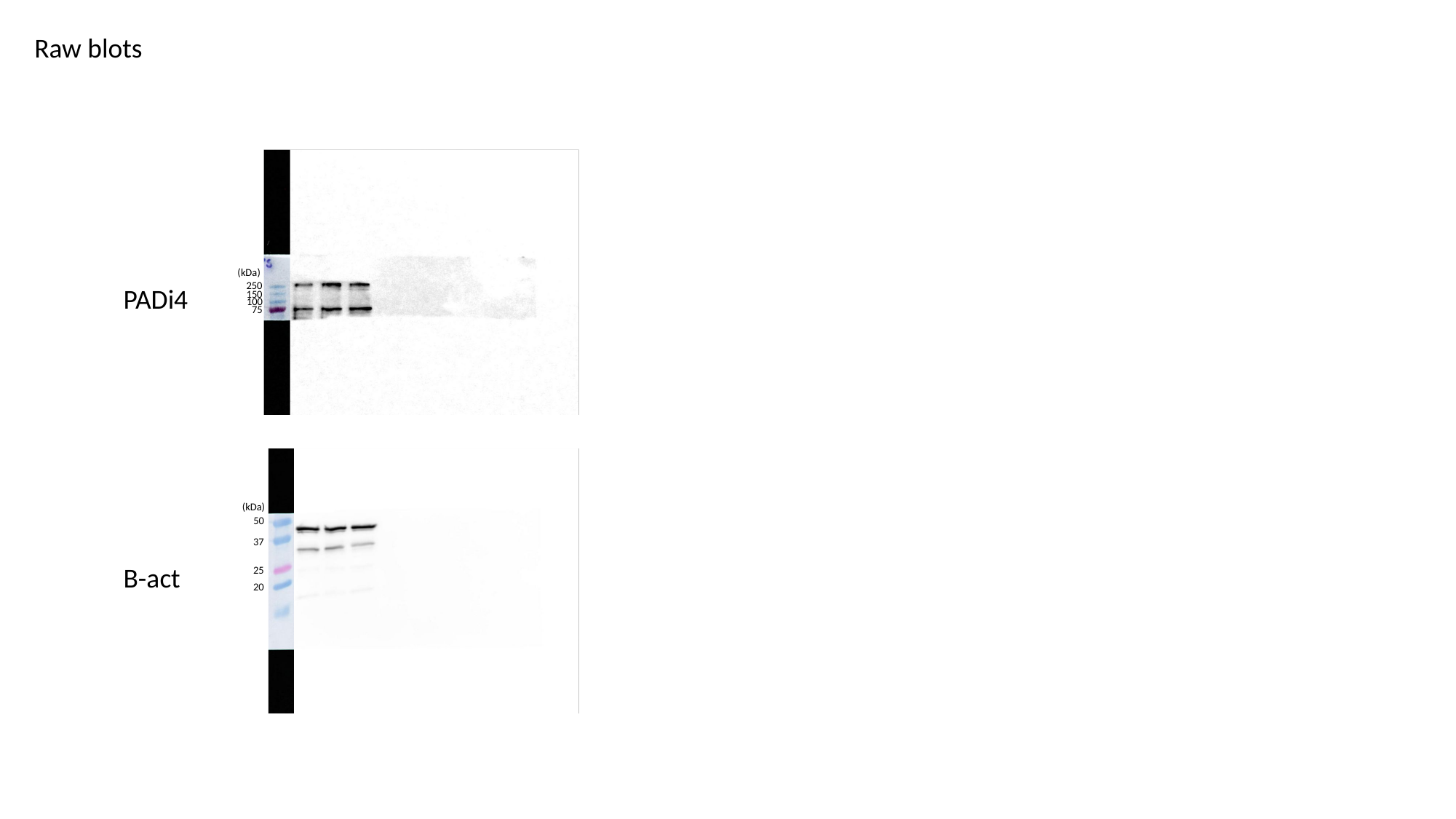

Raw blots
(kDa)
250
PADi4
150
100
75
(kDa)
50
37
B-act
25
20

## Slide 6
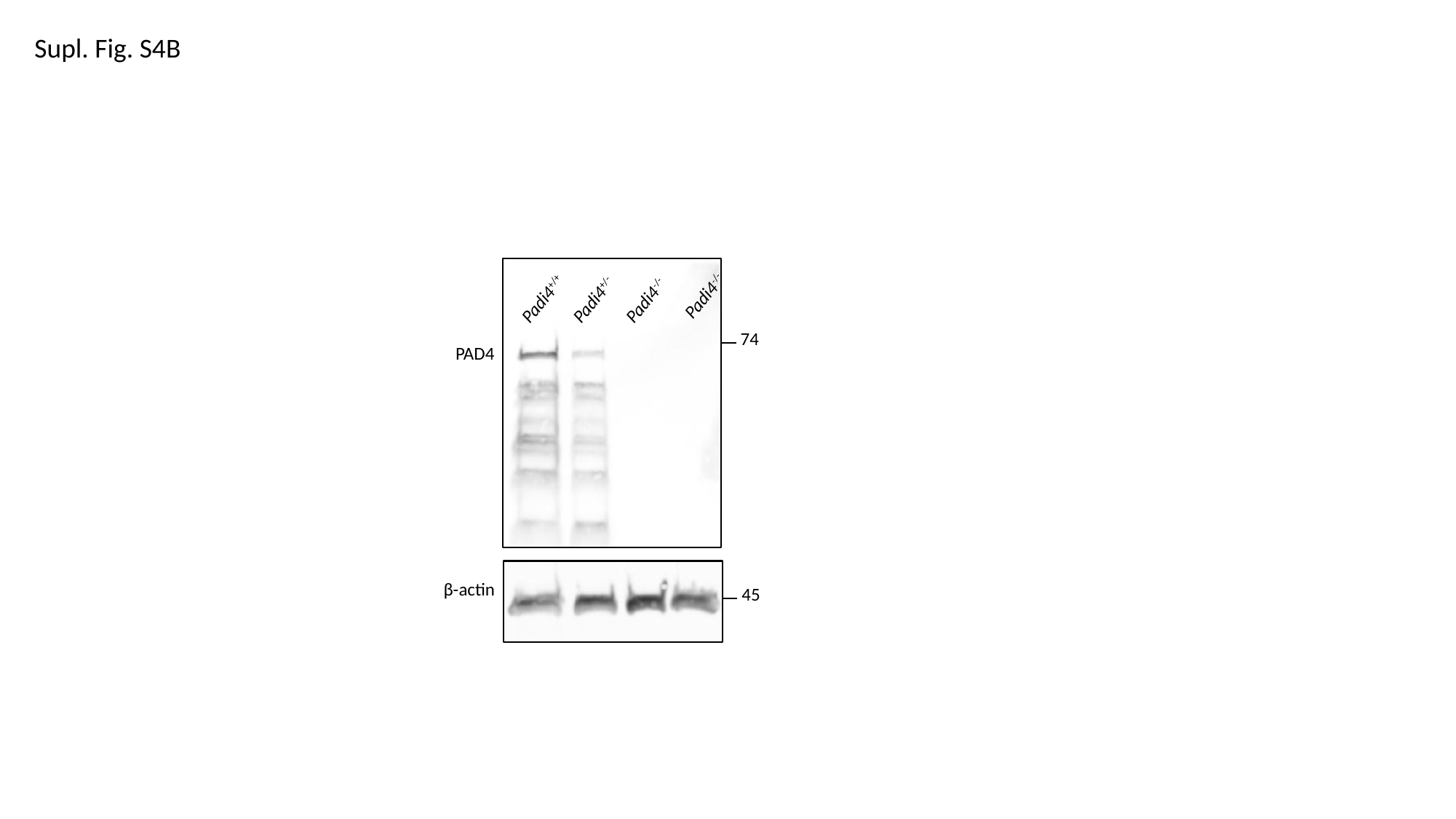

Supl. Fig. S4B
Padi4+/-
Padi4-/-
Padi4-/-
Padi4+/+
74
PAD4
β-actin
45

## Slide 7
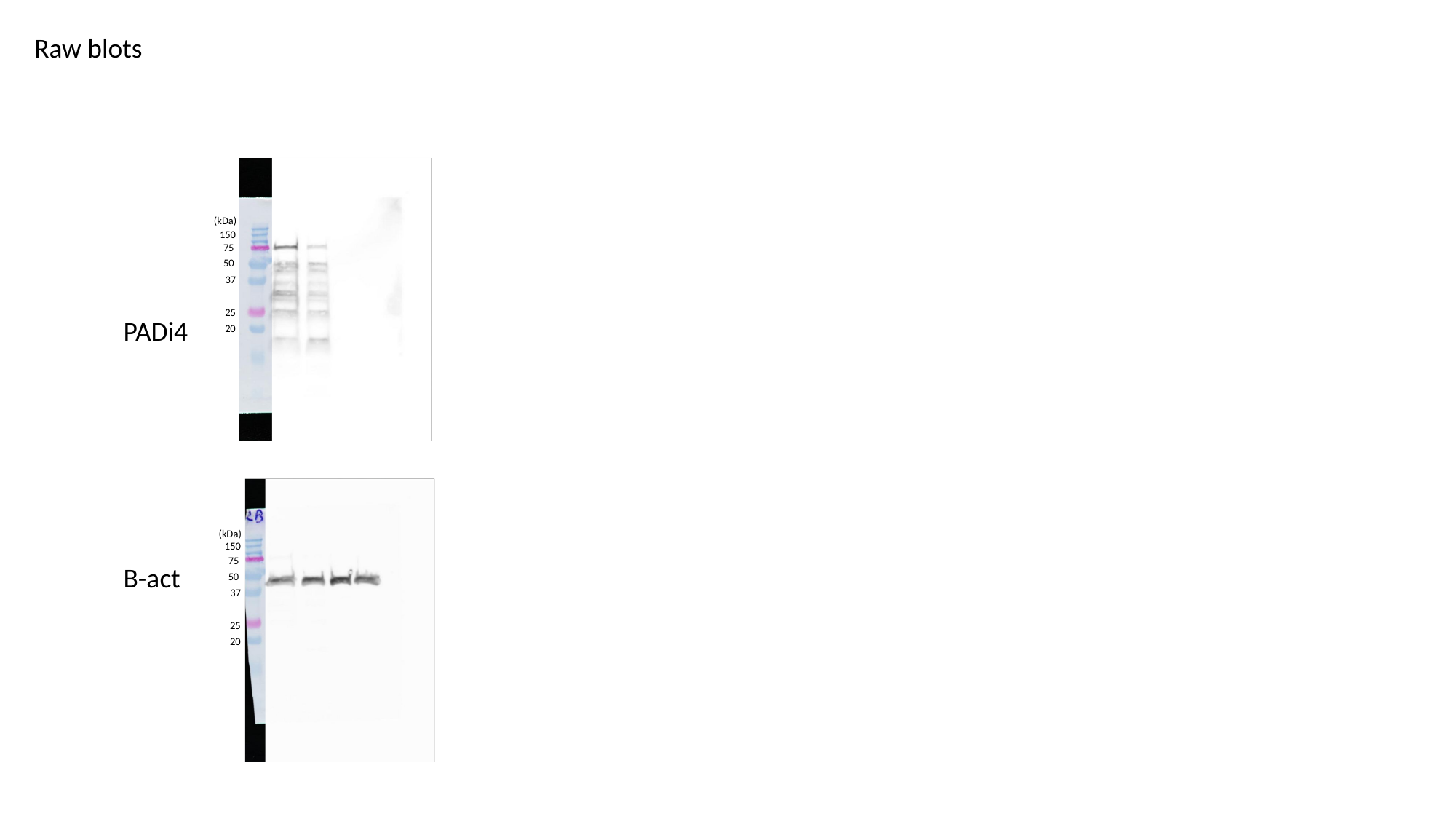

Raw blots
(kDa)
150
75
50
37
25
PADi4
20
(kDa)
150
75
B-act
50
37
25
20
